# *Ab externo* open conjunctiva XEN^®^ 63 μm: a novel surgical alternative in uveitic glaucoma-a case report

**DOI:** 10.1186/s12348-025-00493-7

**Published:** 2025-04-10

**Authors:** Yann Bertolani, Júlia Angrill-Valls, Laura Sánchez-Vela, Eric Kirkegaard-Biosca, Antonio Dou, Nieves Martín-Begué, Marta Castany

**Affiliations:** Department of Ophthalmology, Vall d’Hebron University Hospital, Passeig de la Vall d’Hebron 129, Barcelona, 08035 Spain

**Keywords:** XEN63, Uveitic glaucoma, Hypotonic maculopathy, Decompression retinopathy; minimally invasive glaucoma surgery

## Abstract

**Purpose:**

To describe the efficacy and safety of the *ab externo* open-conjunctiva XEN^®^ 63 μm gel stent in uveitic glaucoma.

**Case presentation:**

A case report of a 15-year-old patient with bilateral uncontrolled uveitic glaucoma secondary to chronic anterior uveitis was presented. Several medical ophthalmological and rheumatological evaluation visits were conducted. Preoperative intraocular pressure was 28 mmHg in the right eye and 32 mmHg in the left eye. She underwent a trabeculectomy in her right eye developing hypotonic maculopathy and decompression retinopathy, requiring surgical revision. Considering the complications endured in the right eye, it was decided to perform an *ab externo* open-conjunctiva XEN^®^ 63 μm implant in her left eye with no postoperative complications. Postoperative course was uneventful with well-controlled intraocular pressure (11 mmHg) and no progression of glaucomatous damage, one year after the procedure. Anterior segment optical coherence tomography evidenced a well-functioning and non-encapsulated bleb along the follow up. Eventually, there were no postoperative uveitic episodes, with 40 mg Adalimumab as a steroid-sparing agent.

**Conclusion:**

Uveitic glaucoma presents both clinical and therapeutic challenges. Traditional surgery such as trabeculectomy may entail postoperative complications such as hypotonic maculopathy and decompression retinopathy. This is the first case reporting the efficacy and safety of *ab externo* open-conjunctiva XEN^®^ 63 μm implant in uveitic glaucoma, highlighting its potential usefulness in such clinical scenarios.

## Introduction

Uveitic glaucoma (UG) is a common and potentially severe complication of uveitis, affecting approximately up to 10% of patients [1]. Herpetic keratouveitis, Fuchs’ heterochromic iridocyclitis and juvenile idiopathic arthritis-associated uveitis are especially prone to ocular hypertension (OHT) and secondary glaucoma. Several mechanisms may contribute and coexist to induce OHT. Closure angle due to peripheral anterior synechiae, steroid-induced OHT and progressive damage of the trabecular meshwork (TM) due to the deposition and accumulation of inflammatory cells may contribute to OHT and eventually to UG [1, 2].

Managing UG may be particularly challenging, requiring balancing intraocular pressure (IOP) control, underlying inflammation, surgical complications and the high heterogeneity between individual cases [3]. Traditionally, filtering surgery (including trabeculectomy (TBT) and non-penetrating deep sclerectomy(NPDS)) and glaucoma drainage devices (GDD) have been the standard of care for UG refractory to medical treatment [1, 3]. In recent years, minimally invasive glaucoma surgery (MIGS) devices have been developed as an alternative to conventional filtering surgery to limit postoperative complications in primary open angle glaucoma (POAG). XEN^®^ 45 μm gel stent (Allergan, Dublin, Ireland) and XEN^®^ 63 μm gel stent (Allergan, Dublin, Ireland) are devices lowering IOP by facilitating aqueous humor outflow from the anterior chamber to the subconjunctival space. Both the *ab externo* with open-conjunctiva (AEO) and *ab interno* with closed-conjunctiva (AIC) approaches have been described for its use [4].

The XEN^®^ 45 μm implant has been successfully employed in the management of UG [5, 6]. However, only one previous study assessed the use of AIC XEN^®^ 63 μm in a case of medically refractory UG [7]. To our knowledge, this is the first case report of a patient with UG successfully managed with AEO XEN^®^ 63 μm implant.

## Case report

A 15-year-old patient with a history of chronic hypertensive non-granulomatous bilateral anterior uveitis with poor IOP and inflammation control was referred to our center. The previous treatment included topical prednisone (twice daily), brimonidine (twice daily), timolol (twice daily) and dorzolamide (twice daily). Additionally, the patient was under oral treatment with daily Prednisone 45 mg and Acetazolamide 125 mg (three times a day).

**Fig. 1:**
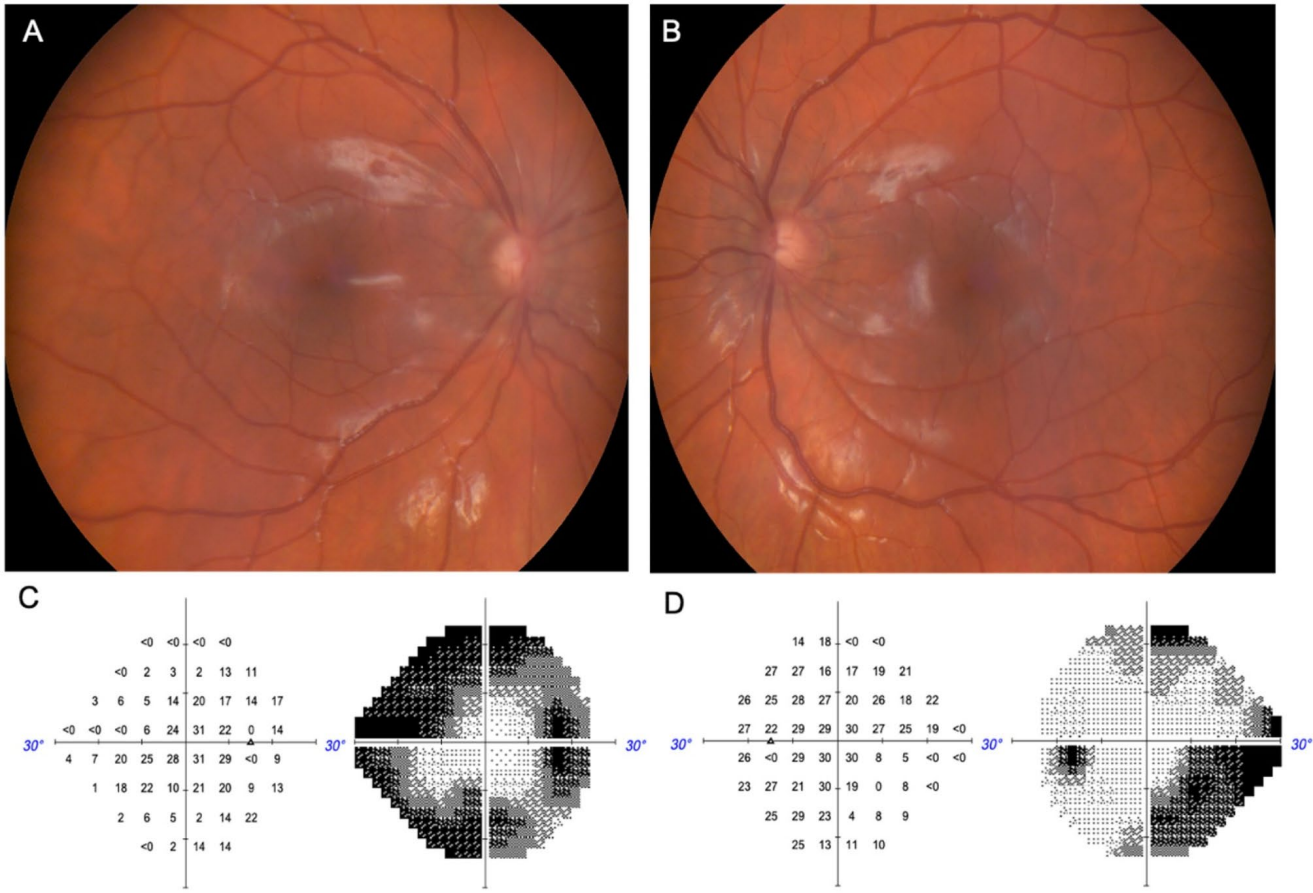
(**A**) OD fundus retinography with a cup-to-disk ratio of 0.8 (**B**) OS fundus retinography with a cup-to-disk ratio of 0.8 (**C**) 24.2º Humphrey visual field test depicting severe glaucoma with superior and inferior arciform scotomas in the OD (**D**) 24.2º Humphrey visual field test depicting severe glaucoma with inferior arciform scotoma in the OS

At our examination, the best-corrected visual acuity (BCVA) was 20/20 and 20/25 in the right eye (OD) and the left eye (OS), respectively. Slit lamp examination depicted the presence of posterior synechiae in OS and the presence of subcapsular cataract in both eyes (OU). Gonioscopy evidence grade IV angle in the four quadrants with mild pigmentation in OU. There were no signs of inflammatory activity in the anterior chamber in OU and IOP was 30 mmHg in OU. The optic nerve cup-to-disk ratio was 0.8 in OU (Fig. 1A and B) and the visual field showed severe bilateral functional loss (Fig. 1C and D).

Suspecting steroid-induced glaucoma, topical and oral corticosteroids were progressively tapered. Moreover, the patient was referred to the Rheumatology department to optimize systemic treatment. No secondary causes of the uveitis were found. Thus, 40 mg Adalimumab was initiated every two weeks for one month, followed by a weekly administration, as a steroid-sparing agent. Likewise, hypotensive treatment was adjusted, increasing Acetazolamide dosage to 250 mg three times a day.

**Fig. 2:**
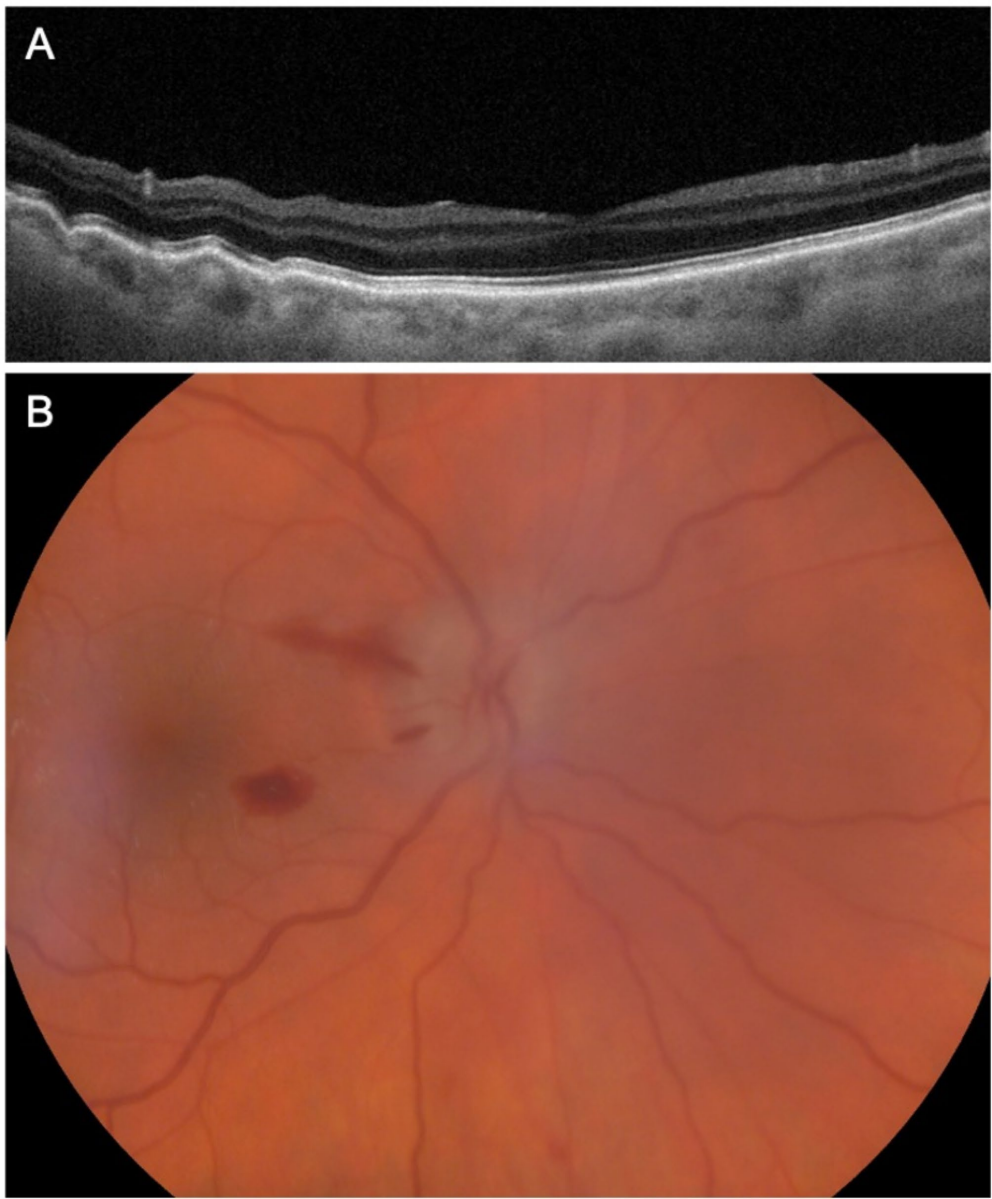
(**A**) Macular OCT of the OD 1 week after TBT in OD featuring hypotonic maculopathy with macular folds (**B**) Decompression retinopathy in the OD encompassing patchy intraretinal peripapillary hemorrhages, splinter hemorrhages and optic nerve edema

Despite the treatment modification and due to uncontrolled IOP (28 mmHg in the OD, 32 mmHg in the OS) associated with severe glaucomatous damage, TBT with MMC 0.02% was performed in the OD. In the early postoperative course, the patient presented hypotony (IOP of 4 mmHg) with hypotonic maculopathy (Fig. 2A) associated with decompression retinopathy, characterized by patchy hemorrhages in the posterior pole and optic nerve edema (Fig. 2B). After 3 weeks with sustained clinically significant hypotony, the previous surgery in the OD had to undergo surgical revision. Given the surgical indication in the OS and the prior complications endured in the OD, an AEO XEN^®^ 63 μm implant was favored over TBT.

Under retrobulbar anesthesia, a superior and temporal dissection of the conjunctiva and Tenon’s capsule with application of mitomycin 0.02% for 2 min were performed. A superior fornix-based conjunctival flap was created followed by a blunt dissection of Tenon’s capsule and conjunctiva with Westcott’s scissors. The scleral tract was carried out using a bent 30G needle, 2.0 mm posterior to the surgical limbus. The XEN was removed from its injector and implanted with toothless forceps through the scleral tract, checking for correct tubular filtration. Subsequently, the Tenon’s capsule and conjunctiva were closed in planes, with placement of sub-Tenon’s Healaflow^®^, as a bleb space modulator.

**Fig. 3:**
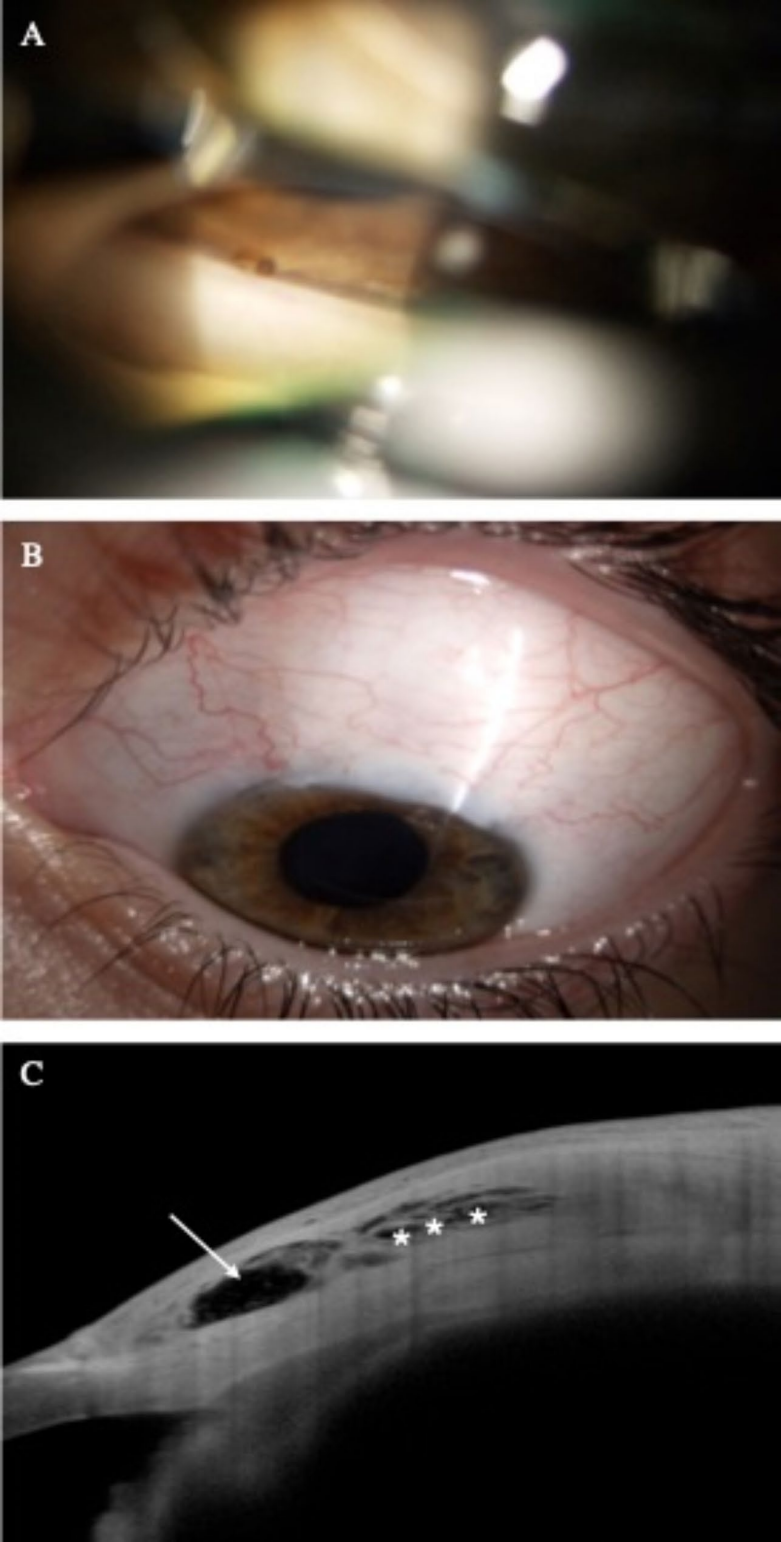
(**A**) Gonioscopy evidencing iris synechiae (arrow) in contact with the XEN^®^ 63 μm with no lumen obstruction, two weeks after the surgery (**B**) Anterior segment photograph depicting a well-functioning XEN^®^ 63 μm bleb 3 weeks after the procedure (**C**) 6 months postoperative AS-OCT higlighting bleb features with a central scleral lake (arrow) and sub-Tenon’s Healaflow ^®^ (asterisks)

 24h after the surgery, the IOP in OS was 12 mmHg with a diffuse and wide filtration bleb, with no Seidel. The anterior chamber was well-formed with no signs of hypothalamia or uveitic flare. The eye fundus showed no signs of hypotonic maculopathy, choroidal detachments nor signs of decompression retinopathy. The correct placement of the device was assessed through gonioscopy. Postoperative topical treatment with descendent phosphate dexamethasone and ciprofloxacin was initiated. An anterior iris synechiae contacting with the XEN^®^ was observed two weeks after the initial procedure (Fig. 3A). However, due to the appropriate bleb functioning and the non-obstruction of the device’s lumen (Fig. 3B), a conservative approach was adopted, and no abnormalities were detected upon the subsequent follow-up.

Successive follow-up showed a correct postoperative course and evolution in both eyes. The IOP remained well controlled with no hypotensive treatment required or adjuvant maneuvers such as needling. At 6 months, the anterior segment optical coherence tomography (AS-OCT) in the OS evidenced a well-formed bleb with persistence of sub-Tenon’s Healaflow^®^ and no signs of fibrosis or bleb-encapsulation (Fig. 3C). One year after the surgery, IOP was 10 and 11 mmHg in OD and OS, respectively. Furthermore, there were no functional nor structural glaucomatous progression in either of the eyes. The BVCA was 20/50 in the OD and 20/25 in the OS, due to the presence of a bilateral subcapsular cataract grade 1, predominantly in the OD.

Eventually, there was a proper control of intraocular inflammation with no uveitic recurrences after one year of weekly Adalimumab 40 mg, as a steroid sparing agent, with adequate tolerance.

## Discussion

The XEN^®^ Gel Stent implant is approved for the surgical treatment of open-angle glaucoma refractory to medical or prior surgical treatment. It is a non-valved implant based on the Hagen-Poiseuille principle to reduce early postoperative hypotonia and associated complications. Currently, there are two XEN^®^ devices approved for its use in the European Union and the USA, the XEN^®^ 45 μm and the XEN^®^ 63 μm.

Both the AEO and AIC approaches for XEN^®^ placement have been described with most studies demonstrating a similar safety profile with similar or even greater effectiveness in the AEO group [8, 9]. Thorough conjunctiva and Tenon dissection, and the placing of bleb filtration modulators may favor long-term effectiveness of the XEN^®^ implant with the AEO approach, reducing the risk of episcleral, Tenon’s capsule fibrosis and late surgical failure [8, 9].

Multiple studies [5, 6] have reported the efficacy and safety of AIC XEN^®^45 in a series of patients with uveitic glaucoma. No uveitic recurrences were described in the postoperative period. Interestingly, the use of AEO XEN^®^45 has not been described in UG. Serrar et al. [7] described the first successful use of AIC XEN^®^ 63 in treating a case of UG after an Ahmed glaucoma valve failure.

In our case, the choice of the AEO approach for the XEN^®^63 was motivated by several factors. Firstly, the postoperative course of the OD with hypotonic maculopathy and decompression retinopathy, requiring a second surgery to revise the previous TBT, justified the use of a safer technique with a lower incidence of postoperative complications. TBT is the most frequently described and employed surgical procedure in the management of uveitic glaucoma. However, it may be associated with a risk of exacerbation of uveitis in up to 12% of cases [10], the appearance of hypotonic maculopathy in up to 28.6% of patients [11] and decompression retinopathy, as in our case [11]. Although hypotonic maculopathy has been described with the XEN® implant, the incidence is lower, affecting only up to 4.6% of cases. Furthermore, in young patients, studies suggest that the risk of secondary needling and surgical failure is higher, probably due to a greater tendency to fibrosis and scaring [12, 13]. Likewise, any revision surgery in case of a postoperative complication may increase the risk of postoperative fibrosis. Additionally, the AEO approach is a minimally invasive technique, limiting intraocular trauma and reducing the risk of postoperative uveitic recurrences.

The XEN^®^ 63 has a lumen of 63 μm, larger than the 45 μm device, implying a greater filtration flow towards the subconjunctival space, providing a greater IOP control in the postoperative period in this patient. This could be especially useful in refractory steroid-induced ocular hypertension, which may be observable in up to 31.5% of patients with uveitis [14].

As a novel surgical device, one of the major limitations of the XEN^®^ is its unknown long-term survival. Hence, the patient may require a surgical revision or another type of surgical procedure in the future. However, the minor surgical trauma associated with XEN^®^, as well as its location in the superior and temporal area, allows for preservation of the superior conjunctiva and Tenon’s capsule for further surgeries.

## Conclusion

The AEO XEN^®^ 63 implant is a safe and effective procedure in patients with open angle uveitic glaucoma. Further studies are required to determine the long-term outcomes of this novel surgical approach.

## Data Availability

No datasets were generated or analysed during the current study.

## Abbreviations

AEO: Ab externo with open-conjunctiva
AIC: Ab interno with closed-conjunctiva
AS-OCT: Anterior segment optical coherence tomography
BCVA: Best-corrected visual acuity
GDD: Glaucoma drainage device
IOP: Intraocular pressure
MIGS: Minimally invasive glaucoma surgery
NPDS: Non-penetrating deep sclerectomy
OHT: Ocular hypertension
OD: Right eye (oculus dexter)
OS: Left eye (oculus sinister)
OU: Both eyes (oculus uterque)
POAG: Primary open-angle glaucoma
TBT: Trabeculectomy
TM: Trabecular meshwork
UG: Uveitic glaucoma
